# “What should I do when I get home?” treatment plan discussion at discharge between specialist physicians and older in-patients: mixed method study

**DOI:** 10.1186/s12913-020-05860-9

**Published:** 2020-11-03

**Authors:** Tahreem Ghazal Siddiqui, Socheat Cheng, Marte Mellingsæter, Ramune Grambaite, Pål Gulbrandsen, Christofer Lundqvist, Jennifer Gerwing

**Affiliations:** 1 Health Services Research Unit, Akershus University Hospital, Lørenskog, Norway; 2 Institute of Clinical Medicine, Campus Ahus, University of Oslo, Oslo, Norway; 3 Geriatric Department, Akershus University Hospital, Lørenskog, Norway; 4 Department of Psychology, Norwegian University of Science and Technology, Trondheim, Norway

**Keywords:** Clinical communication, Discharge, Elderly, Physicians, Medication use

## Abstract

**Background:**

During discharge from hospital, older patients and physicians discuss the plan for managing patients’ health at home. If not followed at home, it can result in poor medication management, readmissions, or other adverse events. Comorbidities, polypharmacy and cognitive impairment may create challenges for older patients. We assessed discharge conversations between older in-patients and physicians for treatment plan activities and medication information, with emphasis on the role of cognitive function in the ongoing conversation.

**Methods:**

We collected 11 videos of discharge consultations, medication lists, and self-reported demographic information from hospitalised patients ≥65 years at the Geriatric department in a general hospital. Mini Mental State Examination score < 25 was classified as low cognitive function. We used microanalysis of face-to-face dialogue to identify and characterise sequences of interaction focused on and distinguishing the treatment plan activities discussed. In addition to descriptive statistics, we used a paired-sample t-test and Mann-Whitney U test for non-parametric data.

**Results:**

Patients’ median age was 85 (range: 71–90);7 were females and 4 males*.* Median of 17 (range: 7 to 23) treatment plan activities were discussed. The proportions of the activities, grouped from a patient perspective, were: 0.40 *my medications*, 0.21 *something the hospital will do for me,* 0.18 *someone I visit away from home,* 0.12 *daily routine* and *0*.09 *someone coming to my home*. Patients spoke less (mean 190.9 words, SD 133.9) during treatment plan activities compared to other topics (mean 759 words, SD 480.4), (*p* = .001). Patients used on average 9.2 (SD 3.1) medications; during the conversations, an average of 4.5 (SD 3.3) were discussed, and side effects discussed on average 1.2 (SD 2.1) times. During treatment plan discussions, patients with lower cognitive function were less responsive and spoke less (mean 116.5 words, SD 40.9), compared to patients with normal cognition (mean 233.4 words, SD 152.4), (*p* = .089).

**Conclusion:**

Physicians and geriatric patients discuss many activities during discharge conversations, mostly focusing on medication use without stating side effects. Cognitive function might play a role in how older patients respond. These results may be useful for an intervention to improve communication between physicians and older hospitalised patients.

**Supplementary Information:**

The online version contains supplementary material available at 10.1186/s12913-020-05860-9.

## Background

Discharge from the hospital can be challenging both for health care workers (with busy wards and time constraints), and for patients with complex needs. Particularly, older patients with comorbidities, polypharmacy and cognitive impairment are vulnerable [1]. After the hospital stay, transferring patients from the hospital to home (back to primary care) may be a challenge [2, 3]. Suboptimal discharge planning and miscommunication between care providers, older patients and informal caregivers may contribute to difficulties [2–4]. In addition, reduced medication safety (e.g., medication errors and non-adherence) is common among older patients [5, 6].

As part of the discharge process, the health care practitioner and patient discuss a treatment plan, which is an intended set of *activities* that should happen to help the *patient to become healthier or stay as healthy as possible, once returning home*. The treatment plan should be tailored to the particular patient with support of a multidisciplinary team [4, 7] and give the patient opportunity to ask questions [8]. The specific treatment plan *activities* can be carried out by the patient, the patient’s general practitioner, the hospital physician, or other health care professionals [9]. Investigating medical consultations can be valuable for improving communication skills among physicians [10].

Previous studies relate improving discharge planning strategies to improved patient outcomes [7, 11]. However, few studies have assessed how information is given during an ongoing discharge conversation between physicians and older patients in clinical practice. A previous study from Norway [9] found that during medical encounters between physicians and patients, one third of the conversation was on treatment plan tasks. However, many tasks still had unclear tasks instructions, and the physician spoke more than the patient. In a study from the United States [12] of medical encounters with primary care physicians, three or fewer clinical decisions were made, often before or during the consultation, mostly by the physicians. Topics of decisions are often around medication, routine tests, follow-up appointments or defining medical problems [13, 14]. To our knowledge, few previous studies have observed conversations in geriatric wards. Thus, characteristics of treatment plans for older in-patients with comorbidities and polypharmacy are unclear.

A previous study [15] reported that 44% of older patients had at least one unnecessary prescribed drug during hospital discharge. The most common unnecessarily prescribed medications were Central Nervous System Depressants (CNSDs), opioids, z-hypnotics, benzodiazepine (BZD) typically used for pain, insomnia and anxiety. However, physicians were better at educating the patients when prescribing new psychiatric and analgesics medications, compared to other medication groups [16]. Studies assessing post-discharge medication knowledge among older patients reported insufficient communication, lack of understanding of side effects [17–19], problems with polypharmacy and purpose of medications [16, 20, 21]. This might be explained by low health literacy and comorbidities [1]. On the other hand, older patients’ use of CNSD medications is common and associated with reduced cognitive function, as demonstrated in cognitive tests related to language (e.g., repetition of easy to complex sentences) [22]. Thus, focusing on associations between communication and such use may contribute towards understanding these challenges.

Cognitive impairment among older patients can lead to difficulties in comprehending discharge instructions [21]. Language difficulties can be an early sign of cognitive impairment among older patients [23], which can go undetected [24]. Studies often use standardised tests to assess language markers [24, 25]. However, conversation patterns are also found to be useful in detecting dementia [26]. Some patterns for patients with mild cognitive impairment are a large vocabulary, compensating for their impairment [27], inconsistency (intraindividual variability), and slow average speech rate [28].

The literature lacks studies on discharge conversations between physicians and older in-patients with complex needs in geriatric wards. The current study aims to address four research questions:

1. *How many treatment plan activities (*i.e.*, what should happen after leaving the hospital) do physicians and older patients discuss?*
2. *What information about medication use do physicians give to the patients before they leave the hospital?*
3. *Can we detect any differences between discharge conversations in patients with and without cognitive difficulties?*
4. *How can we use discharge conversations in geriatric wards to give suggestions for clinical practice?*

## Methods

### Design & settings

The current mixed method study uses 11 video recordings of real-life discharge conversations between physicians and older patients to assess the material in-depth both qualitatively and quantitatively. The data collection is a part of a larger study [22]. We collected data from a somatic geriatric ward of a university hospital. The time period for recruitment was limited from May 2018 to December 2019. The patients were included consecutively, and the discharge conversation was conducted at one of the two last days of the stay. The physicians responsible for discharging the patients were invited to participate in the study.

### Participants and data

The *Inclusion criteria* were: in-patients from the somatic general university hospital departments between the age of 65 and 90 years old*. Exclusion criteria* were: psychosis, brain tumour, traumatic brain injury, stroke, delirium and unable to participate due to medical condition. In addition, patients were *excluded* if they fulfilled the Diagnostic and Statistical Manual of Mental Disorders (DSM)-IV criteria for dementia [29], DSM-V criteria for major neurocognitive disorder [30] and/or moderate to severe depressive episodes according to the International Classification of Diseases (ICD)-10 [31]. We also excluded patients with a Mini Mental State Examination (MMSE) score lower than 21 [32] due to the higher probability that they would have reduced consent ability.

We defined CNSD medication use as using opioids, BZD, Z-hypnotics or a combination of them, regularly for ≥4 weeks prior to hospital admission. Non-use was defined as no CNSD use or sporadic use below the aforementioned threshold.

**Fig. 1:**
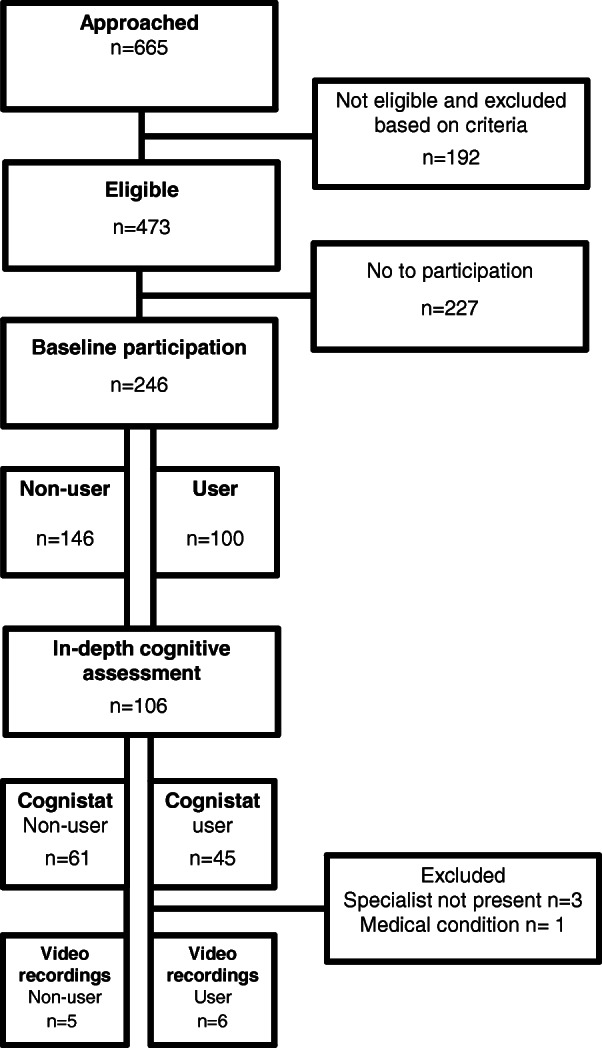
Study participation flow chart at baseline and in-depth sample for current study. Cognistat = The Neurobehavioral Cognitive Status Examination

The flow chart of participants in the study is shown in Fig. 1. At baseline, we collected data from a larger sample. Later, we approached a convenience sample, aiming to do an in-depth assessment focused on treatment plan activities. We obtained informed consent from the patients and the physicians to participate in the current study. We collected 11 video recordings.

#### Video data

Video recordings were done in situ, that is, in the patients’ hospital room or in an examination room in the ward. During the recording, the researcher, physician, and patient were present in the room. The duration of the video recordings was approximately 10–35 min, depending on the length of discharge conversation itself.

#### Other data

The Electronic patient records (EPR) provided the physicians’ discharge notes, patients’ comorbidities (for scoring the Cumulative Illness Rating Score Geriatrics; CIRS-G [33]), the patients’ reason for admission and CNSD use before and after discharge, and the hospitals’ medication list for each patient. In addition, we used patients’ self-reported sociodemographic information.

### Measurements

#### Cognitive tests

We used the MMSE to examine the patients’ cognitive function. The MMSE test is conducted in the wards to assess older patients’ level of global cognitive function [32]. The test takes about 15 mimutes to complete, gives a maximum score of 30 points, and a score < 25 indicates lower cognitive function [34]. The researchers (TGS, SC) or the occupational therapists of the clinical wards conducted the MMSE at the hospital during the patients’ stay.

#### CIRS-G

The CIRS-G total score was used to assess comorbidity among patients. The scale was used to assess disease burden, scoring from no problem to extreme problems (0–4) in major organ systems and neurological, psychiatric, metabolic and musculoskeletal systems [33]. Researcher SC used EPRs to find this information.

### Ethics

Both physicians and patients confirmed participation by written informed consent. Data was stored on a secure hospital server in the communication lab. The data collection and storage were approved by the Akershus University Hospital data protection officer and the Regional Committees for Medical and Health Research Ethics (2016/2289).

### Analyses

#### Statistical analyses

We recorded age, education, length of stay, medication information, MMSE, and the word counts from the conversations as continuous variables. Categorical variables were: gender, living alone, physicians’ level (resident or specialist), patient responsiveness, physicians’ verb choice in the conversations (past/present versus future) tense, and CNSD use/non-use. Results are reported as proportions, mean, standard deviation (SD) median or range (minimum and maximum). For the statistical analyses we used Microsoft Word and Excel version 2013, and IBM SPSS statistics software (IBM Corp. Released 2015. IBM SPSS Statistics for Windows, Version 23.0. Armonk, NY: IBM Corp). We report proportions for responsiveness and past/past versus future analyses due the coding of the variables. Moreover, we conducted t-test for paired samples and Mann-Whitney U test for non-parametric data to examine difference in word count.

#### Video analysis

We used an adapted version of Microanalysis of face-to-face dialogue (MFD) [35]. MFD is a method which allows the researcher to collect a robust, comprehensive set of all instances of a particular phenomenon in a sample of video recorded dialogues: In this case, we collected all instances of physicians and patients discussing the plan for the patient following discharge from the hospital. The results of MFD provide aggregate measures that allow for quantification, including the scope of the phenomenon in the sample and relevant, emergent characteristics for clustering and selecting for closer examination. A secondary (but no less important) product of MFD is the operational definition the researcher creates during the process of analysis, which constitutes both documentation and a tool to apply and adapt in further research.

Before beginning the MFD, the analyst (TGS), who has a background in psychology, transcribed and coded the video into text, using ELAN annotation software [36] and then exporting the transcript to Excel. The transcript facilitated analysis as a searchable, sortable document by assigning a unique number to each utterance. All subsequent analyses used both the transcript of speech and the video, which provided the participants’ audible and visible behaviours that influenced the interpretation of speech. TGS regularly discussed coding decisions with a supervising analyst (JG), a senior researcher with extensive experience analysing video recorded clinical (and non-clinical) interactions. Co-authors (PG and CL) reviewed difficult decisions, and TGS regularly presented examples for discussion at our video analysis seminar, integrating feedback into analytical decisions. Ultimately, TGS and JG ensured decisions followed the operational definitions systematically and consistently.

**Fig. 2:**
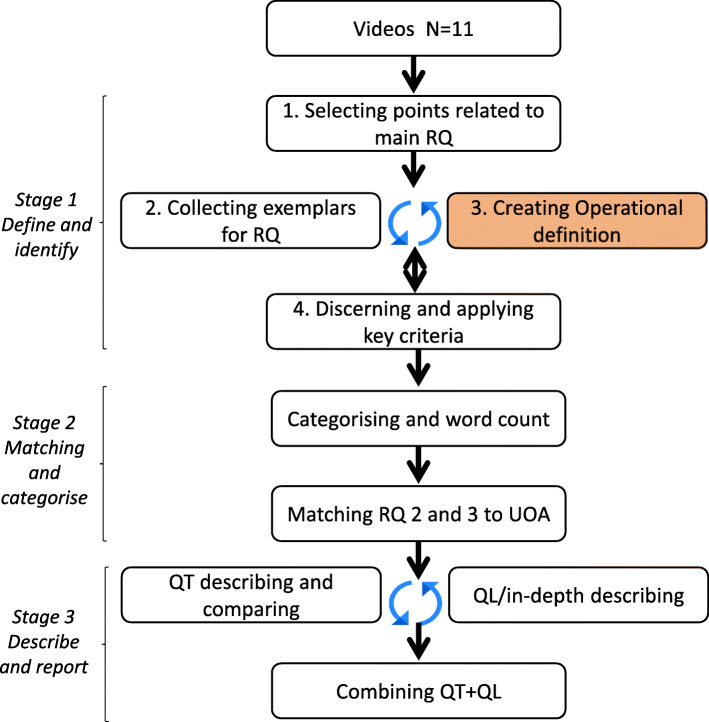
Video analysis by stages. Footnote: RQ = research question, UOA = unit of analysis, QT = quantitative, QL = qualitative

The first stage (**s**ee Fig. 2) of analysis was to identify every instance during which the doctor and patient discussed the treatment plan. Accomplishing this systematically required several steps: (1) The analyst used the main research question (e.g., *How many treatment plan activities do physicians and older patients discuss?*) to determine what behaviours to define and locate (e.g., *patient or physician utterances pertaining to treatment plans*). (2) By collecting and describing the most obvious exemplars, the analyst began the operational definition. (3) Through an iterative process of continuing to collect and explicate examples, the analyst systematically applied and refined the definition, using both unanalysed video and checking for consistency with what was already analysed. (4) Through this process, the analyst was able to discern (and then apply) key criteria to ensure that only the relevant examples were collected. The criteria for treatment plan utterances were threefold: they must be about the patient’s *health* and describe an *action* that someone will do sometime in the *future*. Thus, the product of stage one of analysis was a comprehensive and complete collection of all utterances about the treatment plan; the utterances conveyed multiple individual activities (e.g., start taking a medication, visit the General Practitioner (GP), get a test done).

The second stage of analysis was to propose characteristics along which activities were similar or different. We took a radically patient-centred approach, grouping activities according to what the patient might experience: medications, daily routines, trips outside the home, visitors to the home, and activities for which the patient was not responsible. The analyst found that each activity fit into one of these patient-centred categories. In addition to categorisation, we addressed research questions two and three by comparing how many words were spoken during the parts of the conversation that were about the treatment plan and those that were not.

The final stage of analysis was to filter the collection to analyse some activities in more detail, related to research question one and two. Here, the analyst focused on activities describing medications that patient should take and analysed them according to whether side effects were discussed (related to the second research question). We then focused on comparing patients with higher versus lower cognitive function in more detail related to research question three. This approach gave us the opportunity to report the results both qualitatively and quantitatively.

### Main outcome: qualitative based unit of analysis and detailed definition

We were interested in analysing how the physicians and patients discussed what would happen after the patient was discharged from the hospital. To do this, we began by defining how we would recognise utterances pertaining to *treatment plan activity.* We relied on a previous definition [13], which we adapted to our material. Thus, we defined a *treatment plan utterance as a verbal statement committing to a particular course of clinically relevant action and statement concerning the patient’s health*, we added: *that would involve activities occurring after the patient returned home*. For the detailed definition, we chose to focus on sequences of utterances related to: *health, action and future orientation.* To be included as utterance pertaining to treatment plans, all three criteria were required, both the patient and specialist could initiate the treatment plan activity. More detailed information on the definition is included in the Additional file 1**.**

### Quantitative post hoc exploratory analyses

#### Medication and side effects

As a part of our post hoc exploratory analysis, we examined how many medications were discussed during the treatment plan activities, how many medications were recorded in the EPR, including medications used sporadically/as needed and removed at discharge. In addition, in the total conversation without treatment plan, we assessed utterances for content about physical side effects and cognitive side effects among CNDS users. We counted repeated references to medications at different points of the conversation as distinct activities.

#### Word count

We calculated a word count for both treatment plan and non-treatment plan utterances, to differentiate between treatment plan related talk and other topics. We calculated word counts separately for physicians and patients. We included unclear words, half words and single letters in the word count.

#### Cognitive function and word count

We examined whether cognitive function measures were associated with the number of words spoken during discussions about the treatment plan compared to other topics. We dichotomised the patients in to two groups, MMSE score > 25 indicating higher cognitive function and < 25 lower cognitive function.

#### Responsiveness among patients with high and low cognitive function

We also examined how responsive the patients were during sequences related to the treatment plan. We made a dichotomised score and rated each activity in the patients’ treatment plan. An activity was coded *non-responsive* (“0”) if the patient was silent or only minimally responsive (e.g., “mhm”, “yes”, “I’ll do that”, nodding, or repeating exactly what the doctor had said) and *responsive* (“1”) if the patient did something more (e.g., contributed information, asked questions) [9]. Lastly, we combined responsiveness scores to estimate a proportion for the two groups (lower versus higher cognitive function).

#### Implicit and explicit future related utterances in treatment plan activities

We analysed how the physicians expressed what the patient should do once returning home. We assessed all utterances about the treatment plan, checking whether the physicians were referring to the activities using future implicitly (using past/present verb tense), implying that it should continue in the future, or future explicitly (using utterances with future tense verbs or specific references to future times). We had the impression physicians tended to imply the future in utterances relating to medication activities. To test this impression, we divided our activities into four main groups *(medication activities implicit future* versus *explicit future, and other activities implicit future* versus *explicit future*).

## Results

### Participants

In total, 11 patients and 7 physicians participated in the study. The median age for the patients was 85 years old (range: 71–90), they had median 12 (range: 9–16) years of school and stayed at the hospital for median 6 days (range: 2–20). More females (*n* = 7) than males (*n* = 4) participated, 6 patients used CNSDs, most frequently Z-hypnotics combined with opioids. Six patients lived alone and four had cognitive impairment (MMSE< 25). The reasons for admission among the patients are reported in Table 1**,** and a median of 8 (range: 4–12) comorbidities were found. Among the 7 physicians, 3 were females and 4 males; 4 were residents and 3 were specialist physicians.

**Table 1:** Descriptive information of older patients and physicians

Case	Patient gender	Age at baseline	Education in years	Days of stay	Reason for admission	CNSD	Type of medications	CIRS-G	Lives alone	^a^Cognitive function	^b^Physicians gender	^b^Position at hospital
1	Female	82	12	7	Fall	User	Z-hypnotics	7	Yes	Higher	Female	Resident
2	Female	88	12	6	Anemia	User	Opioid	10	Yes	Lower	Female	Specialist physician
3	Male	76	12	5	Oedema	User	Opioid Z-hypnotics	12	No	Higher	Male	Resident
4	Male	87	12	5	Infection	User	Opioid	7	Yes	Higher	Female	Resident
5	Male	78	9	11	Hypoglycemia	User	Opioid, Z-hypnotics	9	No	Lower	Female	Resident
6	Female	85	12	20	Hip pain, nutrition	User	Opioid, BZD	12	Yes	Lower	Male	Resident
7	Female	80	9	2	Fall	Non-user	N/A	4	No	Higher	Female	Resident
8	Female	87	9	9	Hypoglycemia	Non-user	N/A	6	Yes	Higher	Male	Resident
9	Female	71	16	4	Fever, astma, dyspnoea	Non-user	N/A	10	No	Higher	Male	Specialist physician
10	Male	90	12	12	Oedema	Non-user	N/A	4	Yes	Lower	Male	Specialist physician
11	Female	87	12	5	Hypoglycemia	Non-user	N/A	8	No	Higher	Female	Specialist physician
Median		85.0	12.0	6.0				8				
(Range)		(71–90)	(9–16)	(2–20)				(4–12)				

Footnote: Cognitive function cut-offs, *N/A* Not applicable. User = CNSD use above 4 weeks, Non-users: No CNSD use, or below 4 weeks, *BZD* Benzodiazepine. ^a^Higher = Mini Mental State Examination (MMSE) score > 25, lower = MMSE score ≤ 25. ^b^In total, 7 physicians participated, each physician conducted 1 to 3 discharge conversations. We have reported gender and position at the hospital to illustrate the composition of each dyad

### Main outcome of qualitative features

Physicians and patients discussed a median of 17 distinct activities (range: 7 to 23). Activities clustered into five topics (see examples in Table 2). The proportions of activity topics were: 0.40 was related to *my medications*, 0.21 *something the hospital will do for me,* 0.18 *someone I visit away from home,* 0.12 on *daily routine* and *0*.09 on *someone coming to my home*. Interestingly, we also discovered during observation that the topics in treatment plans were often discussed in a non-systematic way, that is, going back and forth between the topics.

**Table 2:** Topical categories and applied definition with examples

Topics	Examples	Rationale
*My medications*	D: And also if you get fever P: Yes D: Get really ill, then you can take a double dose with Medrol P: Yes, yes, yes D: Because that is important P: Yes, yes	**“Fever and ill”** are related to health, “**you take”** is an action for the patient. The future is “**get/can”.** This topic categorised as *my medication*, because Medrol is a medication the patient should take upon getting a fever.
*Something the hospital will do for me*	D: I am going to write a medical/doctor note to him (GP)	Health related words are **“medical/doctor note”** due to medical information about patient it contains. The action is related to physician: “**I.. write”**, and “**going to”** is related to future. The category is based on the fact that someone from the hospital will write the note for the patient.
*Someone I visit away from home*	D: Hope that the heart failure will adjust P: Yes D: Eh, but that is one of the things, you should control at the GP next week P: Yes, exactly	Health related words are **heart failure,** the, **“you** … **control”** is an action to be performed by the patient**.** The future orientation is**: “next week”.** This category is about visit away from home, as the patient is visiting GP
*Daily routine*	D: Are you driving? P: No, I have not been driving, because I didn’t have any car to drive D: Yes, I want to give you, eh, you shouldn’t drive D: Now that you had the tendency to fall, I would ask you to not do that	The health related words are: **“tendency to fall”**. The patient is doing the action: **“you … driving”**. The future orientation is: **“shouldn’t”** drive (after discharge). This example is then categorised as daily routine, because the driving has been part of the patient’s routine.
*Someone coming to my home*	P: It’s going to be fine, once I get more ointment D: Yes, and the home nurse will help you with that, when you get home P: Yes, that is good, thank you	Health related word is **“ointment”,** the home nurse is doing the action: **“the home nurse will help you”**, in the future: **“when … get home”.** It’s an activity for the nurse, thus the category is someone coming to my home.

Footnote: *D* Doctor, *P* Patient. All the utterances are translated from Norwegian to English

### Quantitative post hoc analyses

#### Medications and side effects

As Table 3 shows, the numbers of medication the patients were using regularly were on average 9.2 (SD 3.1). Only about half (Mean: 4.5, SD: 3.3) were discussed during the discharge consultation. Notably, CNSD related side effects were not discussed for the six patients using CNSDs.

**Table 3:** Number of medications use and side effects

Case	Regularly used medications from EPR (N)	Sporadically/ as needed medications from EPR (N)	Medications removed at discharge from EPR (N)	^**a**^Medications discussed during discharge (N)	^**a**^Physical side effect discussed in conversation (N)	^**a**^CNSD side effects discussed in conversation (N)	CNSD
1	6	0	0	2	0	0	User
2	7	1	2	4	1	0	User
3	12	1	0	3	3	0	User
4	12	2	5	7	0	0	User
5	9	2	0	3	0	0	User
6	11	4	0	4	1	0	User
7	4	0	0	2	0	N/A	Non-user
8	6	0	2	3	0	N/A	Non-user
9	13	5	1	2	0	N/A	Non-user
10	9	0	0	7	1	N/A	Non-user
11	12	2	1	13	7	N/A	Non-user
**Mean**	**9,2**	**1,5**	**1,0**	**4,5**	**1,2**	**0,0**	
**SD**	**3,1**	**1,7**	**1,5**	**3,3**	**2,1**	**0,0**	

Footnote: *N* Number, Central Nervous System Depressants (CNSD), Not applicable (N/A) CNSD not used, Electronic patient record (ERP), ^a^How many medications discussed

#### Word count

The percentage of words in the conversation that focused on the treatment plan was 34%, versus 66% about other topics.

We also compared word count for patients during treatment plan discussions versus other topics. Patients spoke more (mean 759.7 words, SD 480.4) about other topics compared to treatment plan activities (mean 190.9 words, SD 133.9). A repeated-measures t-test found this difference to be significant, (t (10) = 4.84, *p* = .001).

The physicians used on average a similar number of words for both other topics (mean 671.8 words, SD 295.4) and treatment plan activities (mean 530.3 words, SD 216.2). A repeated-measures t-test found this difference to be not significant (t (10) = 1.94, *p* = .081). A sensitivity analysis using non-parametric test did not alter the conclusion.

There was a significant difference between physicians and patients when discussing the treatment plan (*p* < .001) but not when discussing other topics (*p* = .611).

#### Cognitive function and word count

When talking about other topics than treatment plan, patients with higher cognitive function (MMSE> 25) used on average 790.0 words (SD 575.3), while patients with lower cognitive function used 705.0 words (SD 318.0) (Mann-Whitney U test, U = 12.0, *p* = .705).

When talking about the treatment plan, patients with higher cognitive function used on average 233.4 words (SD 152.4), compared to patients with lower cognitive function 116.5 words (SD 40.9) (Mann-Whitney U test, U = 5.0, *p* = .089).

#### Responsiveness among patients with high and low cognitive function

Patients with higher cognitive function were more responsive (proportion 0.59) when talking about their treatment plan, compared to patients with lower cognitive function (proportion 0.33). We did not conduct significance tests on these proportions.

#### Implicit vs explicit future related utterances in treatment plan activities

When the physicians discussed medications the patient should take as part of the treatment plan, a proportion of 0.33 of the time they only implied the future (i.e., using past or present tense verbs). Physicians appeared to do this less so when discussing other activities in treatment plan (proportion 0.11). As above, we did not conduct significance tests on these proportions.

## Discussion

Our main findings indicate that physicians and patients discussed a large number of treatment plan activities; medication was the most discussed activity. However, only half of the medications on the patients’ medication list were a topic of discussion, and side effects were rarely mentioned. Less responsive patients during treatment plan discussions might have cognitive difficulties.

During discharge conversations between physicians and geriatric patients, we observed a median of 17 distinct activities. Our study had higher number of activities compared to previous studies [9, 12, 13], which highlights that for older hospitalised patients, the discharge conversation might be complex and demanding, as well as for the physicians. The activities in our study were focused on five topics and categorised from a patient’s perspective; previous research found that similar topics were discussed during medical encounters [12–14]. However, in our study, the medication related topic dominated. In addition, our study was in line with another Norwegian study that found talk about treatment plans made up one third of the conversation [9]. Patients and physicians used a similar number of words during the overall discharge consultation. This is contrary to what others have found, where the physicians spoke more during the conversation compared to patients [9, 17]. However, during the part of the conversation when the treatment plan was discussed, the patients used fewer words, compared to the rest of the conversation, whereas physicians used almost an equal number of words, regardless of topic. When physicians discussed medications, they sometimes referred to the activities using past or present tense verbs (rather than referring to the future explicitly with verb tense or time references), even though they were describing what the patient should continue to do at home. Others have found similar results [12, 14].

We found that half of the regularly used medications were discussed, and side effects of medications were less discussed during the conversation, as shown by other studies [19–21]. About half of our patients regularly used CNSDs, but they were not informed about side effects associated with CNSDs, as in other studies [15, 18]. The reason why CNSD side effects were not discussed is unclear. We speculate that the hospital physicians assumed that others have informed the patients already (e.g., the GPs), as the CNSDs were introduced before the stay at the hospital.

Patients with lower cognitive function tended to speak less during treatment plan discussions, but the difference compared to patients without cognitive impairment is less clear. However, when discussing other topics, the numbers of words were similar, perhaps the patients were compensating for cognitive impairment by talking more during general topics, as found in another study [27]. The patients with lower cognitive function were less responsive during treatment plan discussions than patients with high cognitive function. Other studies suggest language difficulties among patients with cognitive impairment [22, 26, 28]. Thus, reduced participation may be an indication that patients may have reduced cognitive function, something physicians could consider when providing information.

### Strengths and limitations

A strength of this study is its mixed method study design, as it provides the possibility to examine the data from different perspectives and to connect clinical data with detailed analysis of conversations. Our data are also from actual discharge conversations between physicians and geriatric patients. Thus, the findings are close to clinical practice and might be helpful for offering recommendations to physicians working with older hospitalised patients with similar profiles as ours. Although our sample is small, it included variation: we have male and female patients with normal and impaired cognition and a range in age and education. Similarly, the physicians were seniors and juniors and both females and males.

Microanalysis of face-to-face dialogue requires an intense, detailed decomposition of the observed dialogue. Ideally, we would have liked to recruit a somewhat larger sample; however, this proved to be difficult with older in-patients. In addition, due to the narrow focus of this study, we have not accounted for treatment plan involvement by next of kin, GP, or other health care workers. About half of our patients lived alone, thus managing by themselves with some help from home nurses. Another limitation of our study might be the Hawthorne effect, in the sense that the awareness participants have of being studied might influence their behaviour [37]. However, the participants did not know that analysis would focus on how they discussed specific treatment plan activities. Moreover, it is unlikely that their formulations of such utterances could be influenced by perceptions of social desirability, as could other displays, such as empathy or friendliness. Lastly, the observations made in this study are limited to older patients hospitalised in a geriatric department, but may implicate what happens also in other somatic departments for older patients.

### Implications

As the current study is closely linked to clinical practice, our findings may offer advice to help physicians to communicate more effectively. We propose some strategies which may be helpful in Table 4.

**Table 4:** Suggestions for discharge conversations based on findings

1.	Consider writing down treatment plan activities with the patient	E.g.*, “We can together write down point for point, for what is going to happen when you return home”*
2.	Make sure it is clear that the patient should do (or continue) the activity once returning home, by using future related utterances	*After describing medication decision made at the hospital: e.g., “You should (continue) to do that once you return home”*
3.	Discuss medications explicitly, including which ones remain the same after the hospital stay, what the major side effects are, or where the patient can find or ask about side effects of medications.	E.g.*, “We are going to discuss only the medications that are changed during the stay”* *“The rest remains the same”* *“The main side effects are …, or you can get more information from...”*
4.	To make sure the patients participate more in the treatment plan discussion, ask open questions to check for agreement and understanding	E.g.*, “Tell me what you plan to do when you get home”* *“What is your plan for what you should discuss with the GP?”*
5.	Reduced participation during treatment plan discussion, such as less responsiveness and/or speaking less, might be a sign of cognitive difficulties. Ask open question to check for immediate recall and comprehension level.	E.g.*, “What should you do with your blood pressure medication once returning home?”* *“What is your plan for handling your medications?”*

### Future research

Building on our study, we suggest future research focusing on the structure of presenting information during the discharge conversation. Only half of the patients’ medications were discussed during the discharge; this selection process warrants further examination. Moreover, studies should further investigate the relationship between language and cognitive function in ongoing conversations. On a general level, future studies should examine the function of the discharge conversation more broadly, perhaps it is for the patients to feel secure, have the sense of inclusion, the need to inform or the unchanged traditions in clinical practise. All these questions should be evaluated, also with a larger sample.

## Conclusion

During discharge from hospital, older in-patients receive instruction on a large set of activities that should be performed at home, mostly focused on medication use. However, information on medication side effects is limited, particularly the side effects of CNSDs. In addition, physicians are not always explicit when discussing what should happen when returning home. The level of cognitive function might play a role in a discharge conversation in terms of how responsive the patients are and how much they speak during their treatment plan discussion with physicians. These findings may be useful in improving communication between physicians and hospitalised older patients, perhaps guiding physicians in planning more effective discharge conversations.

## Supplementary Information

**Additional file 1.** Operational definitions of treatment plan activities and other variables used in the study. The additional file includes detailed operational definition of treatment plan activity and of other variables included in analyses, with examples from the discharge conversation.

## Data Availability

The video data that support the findings of this study are closed for public access, due to data protection regulations by Akershus University Hospital data protection officer and the Regional Committees for Medical and Health Research Ethics. However, summarised data are available upon reasonable request from corresponding author, TGS (tahs@ahus.no).

## Abbreviations

CNSDs: Central Nervous System Depressants
BZD: Benzodiazepine
DSM: Diagnostic and Statistical Manual of Mental Disorders
ICD: International Classification of Diseases
MMSE: Mini Mental State Examination
Cognistat: The Neurobehavioral Cognitive Status Examination
EPR: Electronic Patients Records
CIRS-G: Cumulative Illness Rating Score Geriatrics
MFD: Microanalysis of face-to-face dialogue
RQ: Research Question
UOA: Unit of analysis
QT: Quantitative
QL: Qualitative
