# Supplementary material for: “What should I do when I get home?” treatment plan discussion at discharge between specialist physicians and older in-patients: mixed method study
Source: BMC Health Serv Res. 2020 Nov 3;20:1002. doi: 10.1186/s12913-020-05860-9 (PMC7607876; doi:10.1186/s12913-020-05860-9)
Supplement: Supplementary file 1 — Additional file 1. Operational definitions of treatment plan activities and other variables used in the study. The additional file includes detailed operational definition of treatment plan activity and of other variables included in analyses, with examples from the discharge conversation. [file 12913_2020_5860_MOESM1_ESM.docx]

**Additional file 1: Operational definitions of treatment plan activities and other variables used in the study**

| **Category of definition** | **Definition** | **Examples of definition** |
| --- | --- | --- |
| Health definition | The purpose of the *health* aspect of treatment plan is *to become healthier or stay as healthy as possible*. The health can be related to short-term treatment or long-term illness. To be considered as an activity in treatment plan, an utterance had to be related to the patient’s health (see example 1). An utterance that is not related to health can be example 2. | 1. “*And also it’s fine to take an extra* ***pill,*** *if you get a fever, and feel ill”*.  2. *“Yes, how are things at home?”* |
| Action definition | The *action* aspect of treatment plan should be executed by a particular *agent* (the agent can be implicit): the specialist during discharge, the patient, the GPs, the home nursing team, the next-of-kin/family member*.* These agent + action pairs could be identified as a recommendation from anyone mentioned above. The agent included is recognized by terms such as: *GP will*, *you can take,* *we have sent or home nurse will”.*  An example of utterance on action aspect of activity in treatment plan is in example 3. | *3.* “*And also it’s fine to* ***take*** *an extra* *pill, if you get a fever, and feel ill”*. |
| Future  Definition | Future related aspect of treatment plans are identifiable in how activities are described: (1) future oriented verbs, (2) terms referring to future times (e.g.,*“ every day” “continue with this”, “ when you get home”*) (see example 4) or (3) future by implication (past/ present tense verbs describing an activity that happened with a reasonable assumption that it should continue) (example 5, also examples 6 and 7). | *4. “And also it’s fine to take an extra* *pill,* ***if you get*** *a fever, and feel ill”.*  *5. “And you* ***have been*** *put on a new medication named Plavix”.* |
| Enumerating Activities | Our unit of analysis was a particular activity in the treatment plan. One *activity* was represented by one sequence of utterances about the treatment plan that described *a course of action* *that should be done in the future to achieve a particular objective (e.g., the patient should get take a tablet when they have fever)* [9].  For this analysis, if the participants returned to the same activity (see example 6 and 7) (after discussing other topics, due to repetition or questions asked from patient) during the conversation, we counted it as a separate activity. | In the examples below, the physician returns to the same medication (Triatec) in the conversation twice.  First mentioned:  *6. “But we have added a medication named Triatic, it’s the same as Renitec, do you remember it?”*  7. Second mentioned:  *“And we have added the one named triatic”.* |
| Extended treatment plan definition | After we used the operational definition for *treatment plan* to identify which utterances and sequences should be included in our final analysis, we clustered the utterances referring to activities according to the *patients’ point of view.* For example, some utterances referred to activities that involved *someone coming to my home* (e.g., home nurse visit). Others referred to activities the patient would have to accomplish by leaving home “*someone I visit away from home”* (e.g., GP appointment, physiotherapy), or by making changes to how the patient manages his or her *daily routine* (e.g., prevent fall, nutrition).  When there was an overlap of categories, we focused on who is doing the action (agent+action). Example 8 potentially refers both to something the patient must do outside the home (visit GP), and medication the patient must take. By focusing on who is doing the action (the GP will adjust the medication), the topic will be, *Someone I visit away from home*, rather than *my medication.* | *8. “The GP can adjust your medication”* |
| Clarification of grey areas | When the discussion moves from one activity in treatment plan to another, we choose to separate this by examining whether the three criteria was fulfilled for more utterances in a sequence. If it was possible to locate all three criteria in disparate ways in the same sequence, then that would be counted as a separate task. In addition, when the agent was expect to do two tasks (see example 9) we counted that as two separate activities. | 9. *“I am writing a note to the GP, and I am writing a note to the to the home nurse”* |

Footnote: The additional file includes detailed operational definition of treatment plan activity and of other variables included in analyses, with examples from the discharge conversation.
